# Gamification in the Medicine and Surgery course for occupational therapy and physiotherapy undergraduate students

**DOI:** 10.4102/sajp.v82i2.2341

**Published:** 2026-06-12

**Authors:** Amanda Jankowitz, Monique M. Keller

**Affiliations:** 1 eFundanathi, Faculty of Health Sciences, University of the Witwatersrand, Parktown, South Africa; 2 Department of Physiotherapy, Faculty of Health Sciences, University of the Witwatersrand, Parktown, South Africa

**Keywords:** interdisciplinary education, gamification, health professions education, physiotherapy, occupational therapy, clinical practice, medical education

## Abstract

Gamification has emerged as a transformative pedagogical strategy in health sciences education, offering innovative ways to enhance student engagement, motivation and competency development. This state-of-the-art commentary explores the integration of digital badges into the third-year Medicine and Surgery course for occupational therapy and physiotherapy students at a public university in South Africa. Originally introduced to address contextual and educational challenges, digital badges acted as engagement tools aligned with specific learning outcomes and professional competencies. The inclusion of badges was organically developed by lecturers in response to contextual challenges, including inconsistent face-to-face teaching, low engagement, limited interdisciplinary interaction in an online learning environment, and the need to prepare students for clinical practice in resource-constrained settings. Gamification elements were embedded in Moodle, the learning management system, to scaffold learning progression and support the core competencies outlined by the Health Professions Council of South Africa (HPCSA), including therapeutic and professional relationships, professionalism, communication and ethical practice.

**Clinical implications:**

The introduction of gamification and badges may improve clinical, interdisciplinary, and communication skills among undergraduate students. Contextual relevance, inclusive design, and ongoing evaluation incorporating the students’ voices are recommended in sound instructional design through active engagement with gamification practices in health sciences education.

## Introduction

Gamification is increasingly recognised as a pedagogical innovation in health sciences education, particularly for enhancing student engagement, motivation and competency development in clinical training contexts (Hu 2024). As health professions education evolves toward competency-based models, educators are seeking strategies that not only motivate learners but also align with professional standards and prepare students for real-world interprofessional practice, coined in the literature as professional relationships, fostering interprofessional education (IPE) or interdisciplinary education (IDE).

The IPE approach involves collaboration between two or more disciplines in the learning process, with the goal of fostering interprofessional interactions that enhance the practice of each discipline (American Association of Colleges of Nursing n.d.). This approach is grounded in mutual respect and understanding of the value and contributions of each speciality, and typically includes joint goal setting, planning and decision-making.

Through shared educational experiences and collaborative learning, IDE aims to improve patient care and clinical outcomes by integrating knowledge across fields. It serves as a foundational step toward IPE and practice. According to the World Health Organization, IPE occurs when students from two or more professions learn ‘with, from, and about’ each other to enable effective collaboration and improve health outcomes (Oudbier et al. 2024). Therefore, IPE is essential in preparing students for interprofessional clinical practice, which is widely recognised as a cornerstone of high-quality, safe and patient-centred care.

The third-year Medicine and Surgery course at the University of the Witwatersrand. aims to equip occupational therapy and physiotherapy students with foundational medical knowledge, effective communication skills and an understanding of their roles within the healthcare team. During the coronavirus disease 2019 (COVID-19) pandemic, the Medicine and Surgery course transitioned rapidly to an online format, revealing limited opportunities for interdisciplinary learning. Lecturers observed that students lacked opportunities to develop professional competencies, such as communication, ethical reasoning and teamwork. Moreover, significant challenges in students’ self-regulation, motivation and sustained engagement became evident. Without the structure and accountability of face-to-face teaching, many students struggled to plan, monitor and persist with independent learning tasks - patterns widely reported across higher education during emergency remote teaching. Reduced engagement also limited opportunities for interdisciplinary interaction, collaborative learning and the development of core professional competencies such as communication, ethical reasoning and teamwork. These disruptions highlighted a broader concern: students were not only disengaging from immediate course activities but were also missing opportunities to develop the lifelong learning habits required for continuing professional development (CPD) after graduation. In response to these challenges, the teaching team explored gamification as a pedagogical strategy capable of increasing motivation, supporting self-regulated learning behaviours and fostering meaningful participation in online and blended environments.

To address these challenges, gamification was introduced as a pedagogical strategy through the integration of digital badges within Moodle. The badge-based approach was intended to promote early engagement with professional competencies and clarifying expectations regarding communication, professional behaviour and interprofessional clinical practice. These badges represented the successful completion of specific competency-aligned learning activities, rather than formal micro-credentials. Each badge was linked to key Health Professions Council of South Africa (HPCSA) core competencies, including professionalism, collaboration, communication and ethical practice, to signal progress toward the expected level outcomes of the course (Professional Board for Occupational Therapy 2020; Professional Board for Physiotherapy 2023). The badge system did not constitute accredited micro-credentials; instead, it served as an internal motivational and feedback mechanism that provided students with clear indicators of their developing competencies in a flexible, locally relevant manner suited to a resource-constrained teaching context.

This article synthesises relevant literature about the contextual challenges in South African health science education, the course alignment with the HPCSA minimum standards for training, gamification, curriculum innovation outlined in the pedagogical rationale for badge-based gamification, and discusses its alignment with instructional design frameworks, including Technological Pedagogical Content Knowledge (TPACK), and Analysis, Design, Development, Implementation, Evaluation (ADDIE) instructional design model. It concludes with a lecturer reflection, student voice, and recommendations for the sustainable integration of gamification into health sciences curricula, emphasising the importance of contextual relevance, inclusive design, and student voice in curriculum innovation. To situate this pedagogical approach within the health science education realities in South Africa, the following section outlines the contextual and clinical practice challenges that informed the badge-based gamification adoption.

## Contextual clinical practice challenges in South African health sciences education

South Africa’s diverse socio-economic landscape presents significant challenges for health sciences education, particularly in resource-scarce environments (Burdick 2007). In South Africa, the contextual realities of resource-scarce environments present unique challenges for health sciences education. Occupational therapy and physiotherapy students must be equipped to assess and respond to environmental barriers that impact occupational performance and engagement. Traditional content delivery often overlooks these contextual factors, necessitating a more inclusive and locally relevant approach to health sciences and clinical education.

Clinical education for occupational therapy and physiotherapy undergraduate students prepares them to enter the clinical facilities in their third year of study. The transition from foundational theoretical knowledge and application to clinical practice causes anxiety among students working with patients in a new environment (Jindal-Snape 2023). Lack of preparedness, limiting exposure to complex and diverse environments with accompanying high expectations of performance in clinical facilities, was reported by physiotherapy students (Stoikov et al. 2022). In response to these challenges, badge-based gamification was introduced as a structured learning support mechanism aimed at improving engagement and alignment with the HPCSA minimum standards for training.

In response to these challenges, badge-based gamification was introduced as a structured mechanism to increase student engagement with the course content prior to clinical rotations. The intention was not to reduce clinical anxiety or compensate for limited exposure, but rather to address low participation and inconsistent self-directed learning during the preclinical phase. These contextual realities informed the decision to integrate a badge system as a responsive strategy to encourage ongoing engagement, make expectations clearer, and support students in meeting HPCSA-aligned core competencies within the Medicine and Surgery course.

## Health Professions Council of South Africa minimum training standards and core competencies’ alignment

Given the contextual and clinical practice challenges, it was essential that any pedagogical innovation not only enhance student engagement but also remain aligned with national regulatory requirements. This section, therefore, outlines how the course content and badge-based activities were mapped to the HPCSA minimum standards for training and core competencies. Alignment of the Medicine and Surgery course with the HPCSA minimum training standards and core competencies for undergraduate occupational therapy students (Professional Board for Occupational Therapy 2020) and physiotherapy students (Professional Board for Physiotherapy 2023) remains a priority. The HPCSA quality standards and core competencies are listed in the left columns of Table 1 and Table 2. The Medicine and Surgery speciality content areas that align with the HPCSAs standards and competencies are shared in the right columns of Table 1 and Table 2. It was determined that the Medicine and Surgery course adequately aligns with the minimum training standards required for both disciplines.

**TABLE 1:** Health Professions Council of South Africa minimum training standards for occupational therapy matched to the Medicine and Surgery course content.

Core competencies for occupational therapy	Medicine and Surgery course speciality content areas
Professional and ethical behaviour	Orthopaedics with communication task
Therapeutic and professional relationships	Interprofessional discussions and activities in: Communication tasks:
Knowledge supporting an understanding of human body structures and functions. Emphasis on the following systems: Musculoskeletal Neurological Cardiovascular Respiratory systems	Musculoskeletal is covered in: Internal medicine, Plastic surgery and Orthopaedics Neurological is covered in: Neurology Cardiovascular is covered in Cardiothoracic, General surgery and Paediatrics Respiratory is covered in Internal Medicine and Paediatrics
Knowledge supporting an understanding of disease, disorder and trauma as traditionally covered in the following topics: Anatomical pathology Medicine: Internal medicine, neurology, geriatrics, paediatrics, rheumatology, community medicine, pathology Surgery: General surgery, hand, plastic and neurosurgery, ophthalmology and orthopaedics	Anatomical pathology Internal medicine Neurology Paediatrics Rheumatology General surgery Plastic surgery, including hand Orthopaedics

**TABLE 2:** Health Professions Council of South Africa minimum training standards for physiotherapy matched to the Medicine and Surgery course content.

Core competencies for physiotherapy	Medicine and Surgery course speciality content areas
Professional behaviour and practice management Communication (includes education, effective interviewing, counselling)	Orthopaedics
Body structure, organs and systems Cellular and molecular biology Histology	Anatomical pathology
Pathology (aligned with local burden of disease) Anatomical pathology (inflammation, healing and repair; disease on cellular level) Non-communicable diseases (including obesity, diabetes, cardiovascular disease, cancer) Communicable diseases (including HIV and AIDS, TB) Risk factors for illness Risk factors for movement disorders	Anatomical pathology Internal medicine, Cardiothoracics, General surgery, Urology, Obstetrics and Gynaecology, Neurology Paediatrics, Anatomical Pathology All Neurology
Interpretation of special tests and/or investigations Imaging (e.g. X-rays, MRI, fMRI, PET scans, LODOX, ultrasound) Pathology tests	Radiology, Orthopaedics Anatomical Pathology

HIV, human immunodeficiency virus; AIDS, acquired immunodeficiency syndrome; TB, tuberculosis, MRI, magnetic resonance imaging; fMRI, functional magnetic resonance imaging; PET, positron emission tomography scans; LODOX, Low Dose X-ray Imaging System.

According to the HPCSA minimum training standards, the 4-year full-time undergraduate programmes in occupational and physiotherapy may encompass theoretical, practical, clinical and workplace-based elements, utilising various pedagogical approaches, such as face-to-face classroom sessions, blended learning and online methodologies. The curriculum encourages problem-based and inquiry-based learning. Additionally, collaborative group work and interprofessional training are integral components of the course.

Table 1 presents the alignment between the HPCSA minimum training standards for occupational therapy and the Medicine and Surgery course content areas. This mapping ensures that core competencies, such as professional and ethical behaviour, therapeutic and professional relationships, and communication, were integrated into relevant clinical contexts. Professional and ethical behaviour was addressed through communication tasks embedded in orthopaedics, while therapeutic and professional relationships were fostered through interprofessional discussions and collaborative activities. Foundational knowledge of human body structures and functions was emphasised across the musculoskeletal, neurological, cardiovascular and respiratory systems, with corresponding coverage in internal medicine, orthopaedics, neurology, cardiothoracic surgery, general surgery and paediatrics. Additionally, understanding of disease, disorder and trauma was reinforced through topics in anatomical pathology and clinical areas such as internal medicine, neurology, rheumatology, paediatrics and surgical specialities, including general, plastic, hand and orthopaedic surgery.

Outlined in Table 2 is the alignment between the HPCSA core competencies for physiotherapy and the Medicine and Surgery course content areas. This mapping provided the framework for integrating digital badges, ensuring that student engagement activities such as case-based learning, interdisciplinary reflections and collaborative tasks were explicitly linked to professional standards and relevant clinical domains. Digital badges aligned with these HPCSA core competencies (Table 2) serve as tools to acknowledge student engagement in activities mapped to specific Medicine and Surgery content areas. Professional behaviour and practice management were demonstrated through communication-focused tasks embedded in orthopaedics and interdisciplinary reflection activities. Therapeutic and professional relationships were developed through structured collaborative learning activities embedded within the course. Students engaged in asynchronous Padlet discussions, where they worked in small interprofessional groups (occupational therapy [OT] and physiotherapy [PT] students) to analyse and reflect on team roles, scopes of practice and professional responsibilities within cardiothoracic and internal medicine contexts. These discussions were complemented by individual Wakelet reflections, in which students curated key learning artefacts and critically reflected on their own contributions to team-based care.

Clinical knowledge and clinical reasoning were developed through facilitated case-based learning activities. Students were presented with authentic clinical cases focusing on stroke, burns and paediatric conditions. Each case required students to apply theoretical knowledge from internal medicine, neurology and general surgery to interpret patient presentations, identify key problems and propose appropriate management strategies. Cases were discussed collaboratively, with prompts guiding clinical reasoning and decision-making.

Interprofessional communication and collaboration were further strengthened through structured peer-feedback activities. Students reviewed and provided feedback on peers’ case analyses using predefined criteria, encouraging constructive dialogue across disciplines, and promoting shared understanding of professional roles within the healthcare team.

Table 1 and Table 2 illustrate how the Medicine and Surgery course content was aligned with HPCSA standards for both disciplines. Table 1 and Table 2 demonstrate that badge-based activities were not only pedagogically sound but also compliant with national regulatory expectations, reinforcing the credibility and relevance of the gamification strategy. By embedding badges into learning activities that reflect HPCSA competencies, the course design supported students’ transition into clinical practice and promoted the development of professional identity.

## Gamification in health sciences education: Literature synthesis

Gamification is increasingly recognised as a pedagogical strategy that increases student participation and the development of 21st century skills in health sciences education. Gamification refers to the use of game design elements, such as badges, points, leaderboards and challenges in non-game learning environments to increase motivation and engagement (Deterding et al. 2011; Deterding 2012). This is distinct from game-based learning (GBL), which involves the use of full games or simulations as the learning activity itself, where the game environment drives knowledge acquisition, decision-making or skill development. In our study, only gamification and not game-based learning was implemented, with badges used to support engagement within an existing curriculum rather than through a stand-alone educational game.

In physiotherapy and occupational therapy education, gamification has demonstrated positive effects on student participation, creativity and academic performance. Tools such as Kahoot!, Physiotherapy Party and Escape Room formats have been used to foster active learning and collaboration (Ødegaard et al. 2021; Sandoval-Hernández et al. 2023). These strategies promote immediate feedback, problem-solving and clinical reasoning. These skills are essential for professional practice.

Gamification also benefits from integration into learning management systems, such as Moodle and Canvas, allowing lecturers to embed interactive modules, simulations and assessments directly into the curriculum. Advanced technologies like Augmented Reality, Virtual Reality and mobile applications further expand the possibilities for immersive learning experiences (Stathakarou et al. 2024).

Despite its promise, literature highlights the importance of thoughtful design and contextual relevance. Factors such as learner characteristics, novelty effects and institutional support influence the success of gamification initiatives. Moreover, there is a need for more high-quality research on the long-term impact of gamification on clinical preparedness and workplace readiness in health sciences education (Noyes et al. 2020).

In resource-constrained environments, gamification can complement traditional teaching methods by providing meaningful engagement opportunities without requiring extensive technological infrastructure. When digital badges are aligned with professional competencies and embedded into inclusive course design, they function as a mechanism for recognising learning achievement, scaffolding skill development, and making progress toward clinical competence more visible to students. Building on this literature, the next section describes how digital badges were operationalised within the Medicine and Surgery course, detailing their alignment with course outcomes, professional competencies and learning activities, as well as the criteria and processes through which badges were earned and awarded.

## Curriculum innovation: Digital badges and instructional content in the Medicine and Surgery course

Digital badges have emerged as a micro-credentialing tool that supports competency-based education. They offer granular recognition of achievement and can be aligned with specific learning outcomes and professional standards (Perkins & Pryor 2025). Studies show that badges enhance motivation and confidence, especially when embedded meaningfully into course design (Fajiculay et al. 2017; Truskowska, Emmett & Guerandel 2023).

Digital badges were embedded within the Moodle learning management system as tools aligned with HPCSA core competencies, including communication, collaboration, clinical reasoning, ethical practice and teamwork. They served as formative assessment tools and motivational markers, awarded for completing activities that scaffolded learning and supported professional identity formation. Badge-linked tasks included clinical case studies on cardiothoracic, stroke, burns and hand injury scenarios to promote application of theoretical knowledge; professional communication exercises in orthopaedics to foster ethical and interdisciplinary dialogue; and terminology and equipment modules to prepare students for real-world healthcare environments. Participation badges recognised engagement in interactive tasks such as Padlet reflections, Wakelet contributions, and equipment introductions, emphasising completion and active involvement rather than performance-based grading. This integrated approach aimed to guide learners through complex content, encourage collaboration, and enhance readiness for clinical practice in resource-constrained settings. Student feedback played a pivotal role in evaluating the badge system. Learners reported that badge-linked activities were motivating and meaningful, particularly those involving interprofessional collaboration. They appreciated opportunities to engage with peers across disciplines and found badges helpful for tracking progress. However, students also expressed concerns about the volume of badge-related tasks, which limited their ability to engage deeply with each activity. This feedback informed the course designers of iterative improvements, including reducing the number of badges and streamlining activities in subsequent course iterations.

## Pedagogical frameworks for gamification

While the initial implementation of digital badges in the Medicine and Surgery course was not guided by a formal instructional design framework, lecturers recognised the need to retrospectively align the inclusion of badges with established pedagogical models to ensure sustainability and scalability. Three complementary frameworks, TPACK and the ADDIE were identified as suitable for guiding future iterations of gamified learning in health sciences education.

Technological Pedagogical Content Knowledge offers a holistic model for integrating technology in ways that are pedagogically sound and content relevant. In the context of gamification, TPACK supports the alignment of badge-based activities with clinical learning outcomes, ensuring that technology enhances rather than distracts from educational goals (Elas, Majid & Narasuman 2019).

Analysis, Design, Development, Implementation, Evaluation provides a structured process for instructional design. Although the badge system emerged from practice-based insight, ADDIE can be applied retrospectively to evaluate its effectiveness and prospectively to guide the refinement of gamified components. This includes analysing student engagement data, redesigning badge criteria and implementing feedback loops for continuous improvement (Lu & Sides 2022).

Together, these frameworks offer a robust foundation for designing, evaluating and sustaining gamified learning experiences that are responsive to both pedagogical goals and the contextual realities of South African health sciences education. Their application ensures that digital badges are not merely motivational tools but are embedded within a coherent and evidence-informed curriculum strategy.

## Discussion

The aim of this health sciences practice-informed commentary was to explore the integration of digital badges into a Medicine and Surgery course for third-year undergraduate occupational therapy and physiotherapy students, in response to challenges in contextual clinical education, regulatory requirements and the need to foster interdisciplinary competencies. The discussion that follows integrates pedagogical theory, student feedback and lecturer reflection to evaluate the effectiveness of the approach.

### Pedagogical rationale

Research indicates that digital badges can enhance motivation and participation, particularly when students perceive them as relevant and desirable (Dowling-Hetherington & Glowatz 2017; Higashi & Schunn 2020). In online and blended learning environments, badges can encourage students to interact more with course materials and participate in learning activities (Newby & Cheng 2020). However, the literature also notes mixed results – some studies report negligible or no impact, and others highlight adverse effects when badges are poorly integrated or perceived as extrinsic motivators (Morris et al. 2019). Strategic implementation is therefore critical to ensure badges reinforce core competencies rather than serve as superficial incentives.

### Theoretical foundation

Although badges were introduced through practice-based decision-making rather than formal design models, their effectiveness can be strengthened by applying established instructional design frameworks, such as ADDIE and TPACK (Elas et al. 2019; Lu & Sides 2022). These frameworks ensure that badges are systematically linked to learning objectives, embedded within course design, and aligned with broader curricular and professional standards. For example, ADDIE provides a structured process for analysis, design, development, implementation and evaluation, while TPACK supports the integration of technology in ways that are pedagogically sound and content relevant. These pedagogical considerations were further delineated by student feedback, providing insight into how badge-based learning was experienced in practice.

### Student voice and course evaluation

As discussed earlier, student feedback informed the iterative course improvements. Incorporating student voice aligns with best practices in curriculum design and ensures that gamification strategies remain responsive to learner needs (Marder et al. 2022; Steyn, Davies & Sambo 2019).

### Lecturer reflections and student voice

As a practice-informed commentary, this paper underscores both student voice and lecturer’s reflections as key sources of insight into the effectiveness of the badge-based approach. Informal lecturers’ observations provided valuable information during the reimagining of the Medicine and Surgery course, revealing students were disengaged in the online format and struggled to connect course content with clinical practice. In response, badge-based activities were introduced to scaffold learning and promote interdisciplinary collaboration.

Lecturers reflected positively on the impact of the badge system, noting that it helped guide students through complex course materials and encouraged participation in professional discussion tasks. The integration of platforms such as Padlet and Wakelet enabled students to reflect on team roles and clinical scenarios, fostering deeper engagement and supporting the development of professional identity and communication skills.

From the student perspective, feedback indicated that badge-based activities were both motivating and meaningful, particularly those involving interprofessional collaboration. Students appreciated the opportunity to engage with peers across disciplines and found that the badges helped them track their progress and clarify expectations. However, some students reported that the volume of badge-linked tasks was overwhelming, which led to time constraints and limited their ability to engage deeply with each activity.

This feedback was instrumental in refining the course design. In subsequent iterations, the number of badges was reduced, and activities were streamlined to improve the depth and quality of engagement. The inclusion of student voice in course evaluation was essential to this iterative improvement process. Lecturers used both informal feedback and structured reflection tasks to assess the effectiveness of badge-linked learning. This participatory approach aligns with best practices in curriculum design and underscores the importance of co-creating learning experiences that are inclusive, responsive and grounded in the realities of clinical education.

Considered collectively, lecturer reflections, student feedback and the alignment with instructional design frameworks suggest that badge-based gamification can meaningfully support competency development when thoughtfully integrated and iteratively refined. Overall, this commentary demonstrates how badge-based gamification can emerge organically in response to contextual challenges, and how reflective alignment with pedagogical frameworks can enhance coherence and sustainability.

### Recommendations

**FIGURE 1:**
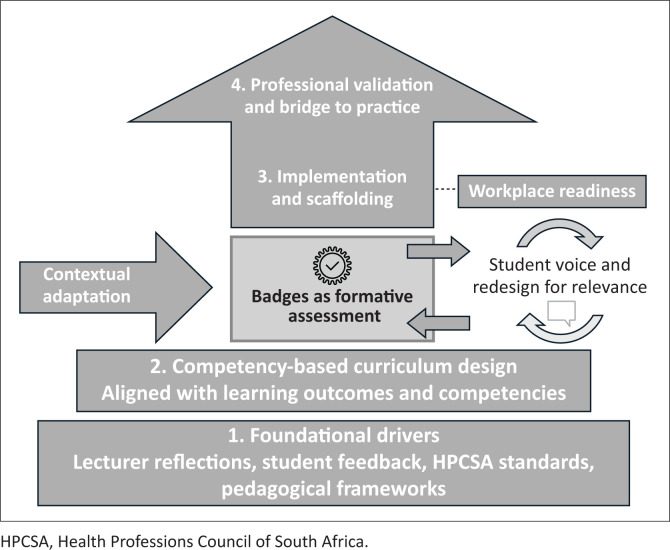
Digital badge value chain for health sciences education.

First of all, gamification should be embedded within competency-based curriculum design (Mukurunge et al. 2020). Digital badges must be intentionally aligned with specific learning outcomes and professional competencies, such as communication, ethical practice and interdisciplinary collaboration. This ensures that gamified elements reinforce, rather than distract from, clinical education goals (Noyes et al. 2020) (see Figure 1 for an overview of the digital badge value chain).

To support pedagogical coherence and meaningful student engagement, instructional design frameworks like TPACK and ADDIE should guide the development, implementation and evaluation of gamified learning experiences. These models help lecturers integrate technology in ways that are both contextually relevant and educationally sound (Elas et al. 2019).

Digital badges can serve as low-cost, scalable tools for formative assessment and micro-credentialing. When thoughtfully designed, they motivate learners, scaffold complex content, and support the development of professional identity (Roy & Clark 2019). Their use should be strategic, focusing on competencies that are critical for clinical preparedness and workplace readiness (Newby & Cheng 2020; Perkins & Pryor 2025).

Incorporating student voice into course evaluation and redesign is essential. Iterative feedback from students should inform the scope, pacing and relevance of badge-based activities. This participatory approach enhances inclusivity and responsiveness, ensuring that gamification strategies remain grounded in learner needs and real-world clinical demands.

To extend the value of digital badges beyond the classroom, institutions should engage clinical supervisors and employers. Raising awareness about the competencies signalled by badges can help stakeholders recognise their relevance and utility in clinical settings, thereby strengthening the bridge between academic learning and professional practice.

Gamification strategies must also be adapted to local contexts and resource constraints. In low-resource environments, lecturers are encouraged to innovate organically while remaining grounded in evidence-informed practice. Flexibility and contextual sensitivity are key to ensuring that gamification enhances, rather than complicating the learning experience (Hung 2017; Wolf et al. 2018; Yildirim 2017).

Finally, further research is needed to explore the long-term impact of digital badges on student performance, clinical preparedness and professional development. Studies should also examine institutional and patient-level outcomes to validate the broader effectiveness of gamified strategies in health sciences education.

## Conclusion

This commentary examined the integration of digital badges as a gamification strategy within the Medicine and Surgery course for third-year occupational therapy and physiotherapy students at a South African university. Situated within the realities of the health sciences education context, the intervention responded to persistent challenges related to student engagement, fragmented learning experiences and limited interdisciplinary interaction.

Introduced to address low engagement and limited interdisciplinary interaction, badges were aligned with HPCSA minimum standards for training and core competencies, supporting communication, collaboration and professional identity formation, thereby supporting the development of communication, collaboration, ethical practice and professional identity formation across disciplines (Roy & Clark 2019).

Although initially implemented without a formal framework, the reflective evaluation of the intervention highlighted the need for greater pedagogical alignment and intentional design (Elas et al. 2019; Lu & Sides 2022). Lecturer reflections and student feedback highlighted badges as motivating and meaningful, though concerns about task volume led to iterative refinements that improved engagement, demonstrating the value of ongoing responsive course design and evidence-informed adjustment.

Gamification, when contextually grounded and strategically designed, offers a powerful tool for competency-based education in resource-constrained environments (Hung 2017; Wolf et al. 2018). Future research should explore the long-term impact of badge-linked learning on clinical preparedness, professional identity and workplace readiness, using mixed-methods approaches to capture both performance outcomes and learner experiences (Noyes et al. 2020; Perkins & Pryor 2025). Such work could further contribute to the evidence base on scalable, low-cost innovations that enhance equity and quality in health sciences education.
